# Experimental evidence that shear bands in metallic glasses nucleate like cracks

**DOI:** 10.1038/s41598-022-22548-8

**Published:** 2022-11-02

**Authors:** Alan A. Long, Wendelin J. Wright, Xiaojun Gu, Anna Thackray, Mayisha Nakib, Jonathan T. Uhl, Karin A. Dahmen

**Affiliations:** 1 Department of Physics, Institute of Condensed Matter Theory, University of Illinois at Urbana Champaign, 1110 West Green Street, Urbana, IL 61801 USA; 2 Department of Mechanical Engineering, One Dent Drive, Bucknell University, Lewisburg, PA 17837 USA; 3 Department of Chemical Engineering, One Dent Drive, Bucknell University, Lewisburg, PA 17837 USA

**Keywords:** Phase transitions and critical phenomena, Glasses, Glasses, Mechanical properties, Metals and alloys

## Abstract

Highly time-resolved mechanical measurements, modeling, and simulations show that large shear bands in bulk metallic glasses nucleate in a manner similar to cracks. When small slips reach a nucleation size, the dynamics changes and the shear band rapidly grows to span the entire sample. Smaller nucleation sizes imply lower ductility. Ductility can be increased by increasing the nucleation size relative to the maximum (“cutoff”) shear band size at the upper edge of the power law scaling range of their size distribution. This can be achieved in three ways: (1) by increasing the nucleation size beyond this cutoff size of the shear bands, (2) by keeping all shear bands smaller than the nucleation size, or (3) by choosing a sample size smaller than the nucleation size. The discussed methods can also be used to rapidly order metallic glasses according to ductility.

## Introduction

Slowly sheared bulk metallic glasses (BMGs)^1–4^ and many other materials^5–10^ deform intermittently, via shear bands. An infamous example of intermittent behavior is earthquakes. There, the slowly increasing stress triggers local slips when the local stress exceeds a stress threshold to sliding. In physics, the associated serrations in the stress–strain curves (i.e. sudden load drops or displacement jumps) are called slips or “slip avalanches,” regardless of whether they occur in crystals or amorphous materials. For applications these slips are undesirable because they make the deformation difficult to control. On tectonic scales they can be disastrous, because they sometimes cause catastrophic destruction. In order to achieve smoother deformations, it is important to understand how these slips initiate, especially the largest slips because they constitute the largest deviations from smooth deformation.

How exactly large slips develop is not yet fully understood: models with material weakening during the slips suggest a nucleation mechanism. They are not cracks and yet they seem to be very similar to cracks^11–16^. They can be used to distinguish between brittle and ductile materials – models of ductile materials without any weakening point to percolation, finite size effects, or other crossover phenomena. Initial evidence of slip nucleation in brittle BMGs was seen in recent nanoindentation experiments^17^. The notion of nucleation was further supported by recent simulations^12^.

Here, we show the results of high-resolution experiments from compression testing that are able to temporally resolve the detailed dynamics of the actual nucleation phase. Using a simple model we show from the experimental data how this nucleation initiates and evolves into catastrophic slips when it reaches a critical size. It then becomes clear how dynamic properties such as the measured critical nucleation size can be used to extract information about the underlying deformation mechanism including the amount of dilation and associated weakening, and the closely related (relative) brittleness of the material.

Even though our experiments focus on BMGs, the results should be transferrable to many other systems. The reason is that very few conditions are needed to establish this kind of nucleation. It only requires the combination of coupled threshold processes with some kind of temporal weakening. Therefore, similar nucleation mechanisms are expected for other systems that show stick slip behavior and a weakening mechanism, regardless of scale. Well known examples include crystals with dynamic strain aging, slowly sheared granular materials, friction, and earthquakes^10,18^.

## Bulk metallic glasses

Even though BMGs have many excellent properties for applications, the limited ductility of BMGs remains an obstacle to their widespread use in load-bearing components. Unlike crystalline metals, monolithic metallic glasses do not work harden. The BMGs that contain crystalline second phases do show some hardening, but the amorphous matrix softens when plastically deformed. This *weakening* during deformation leads to the formation of shear bands. Ultimately one or more shear bands^11,19,20^ propagate (i.e. slip) and can cause catastrophic failure even at relatively low strains.

Shear bands are regions of intense deformation, typically about 20 nm thick^11,21^ in metallic glasses. They are the primary mechanism of plasticity in BMGs, even including specimens smaller than 100 nm^22,23^. Two types of shear bands form in metallic glasses: “small” (i.e. microscopic) shear bands that are limited in extent and “large” (macroscopic) shear bands that, in uniaxial tension and compression, propagate from one side of the specimen to the other, i.e. they span the system. Small shear bands make it possible for plastic strain to accumulate; they allow a BMG to “bend before it breaks.” On the other hand, large shear bands can cause failure because they are system spanning. The key to making more ductile BMGs then is to promote the propagation of the small shear bands but limit the propagation of the large ones through engineering of the microstructure. Before we can engineer the microstructure to resist weakening, however, we must understand the mechanism by which it occurs. Here we demonstrate the mechanism by which large shear bands form.

## Experiments

We employed constant-displacement-rate uniaxial compression experiments (10^–4^ s^–1^ strain rate) on millimeter-size specimens with a composition of Zr_45_Hf_12_Nb_5_Cu_15.4_Ni_12.6_Al_10_. This composition has been previously studied^1,2,7^ and shows shear-banding behavior similar to other BMGs^24^ and atomistic simulation^12^. It is notable, however, within the family of Zr-based BMGs for its excellent glass forming ability and a ductility that makes it a viable candidate for studying shear band statistics^25,26^. The load data from a 60 kN piezoelectric load cell were acquired at a rate of 100 kHz. The details of the experimental setup are the same as in^1,26,27^.

**Figure 1:**
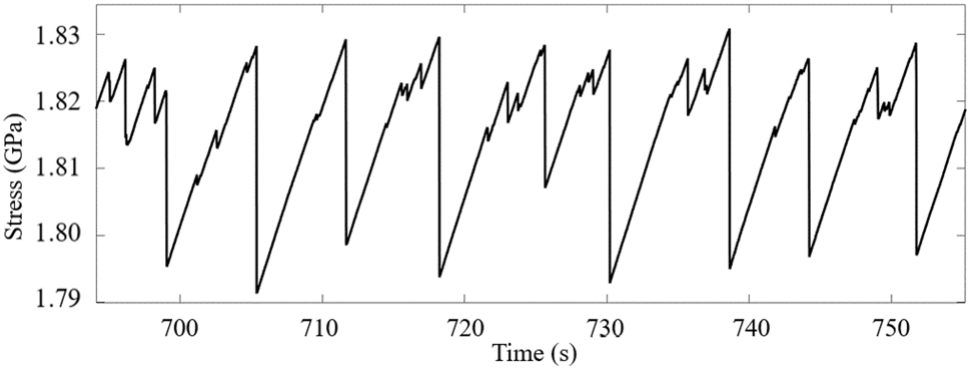
Experiment: The stress-time curve for uniaxial compression of a bulk metallic glass of composition Zr_45_Hf_12_Nb_5_Cu_15.4_Ni_12.6_Al_10_ showing serrated flow. The sample is rectangular with a cross-sectional area of 2 mm by 1.5 mm and a length of 6 mm. The rapid decreases in stress are the avalanches. All visible avalanches are large; however, this behavior occurs over many orders of magnitude of stress drop size. “Small” avalanches are much smaller than is visible in this plot.

During uniaxial constant-displacement-rate compression of a monolithic metallic glass, the specimen loads elastically up to the yield stress, near which point serrated flow behavior commences, as in Fig. 1. During serrated flow, the propagation of each shear band is observed as a stress drop, and the corresponding displacement increments for the largest serrations are on the order of microns. Microscopically small slips are not visible at the scale of stress–strain curves; their displacement increments are on the order of tens to hundreds of nanometers. This behavior in Fig. 1 is typical for a monolithic metallic glass although the specimen shows unusually large plasticity due to the presence of a pore on its fracture surface, which serves as a starting point for many small serrations^1,2^.

Here we answer the questions of why and how large slips form and what we can learn from their dynamics about the deformation mechanism and the material properties. We consider four different mechanisms for the formation of large slips. We eliminate the first three based on experimental observations and provide experimental evidence for the fourth. The discussed four possible scenarios are:

1. *Percolation:* small, localized shear events link to form a large shear band that when fully developed propagates across the specimen.
2. *Finite machine stiffness:* if the load frame is not sufficiently stiff, then small shear bands are driven to be large shear bands by absorbing the elastic energy released by the load frame as the stress drops.
3. *Finite size effects*: the specimen is sufficiently small that all slips that would be microscopic in a larger specimen appear as system spanning for small specimens.
4. *Nucleation*: beyond a certain critical slip size (referred to here as the nucleation size), the slip dynamics changes. The shear band accelerates rapidly and spans the system.

We first discuss the experimental data and then compare them to each of these cases in turn.

## Experimental data

**Figure 2:**
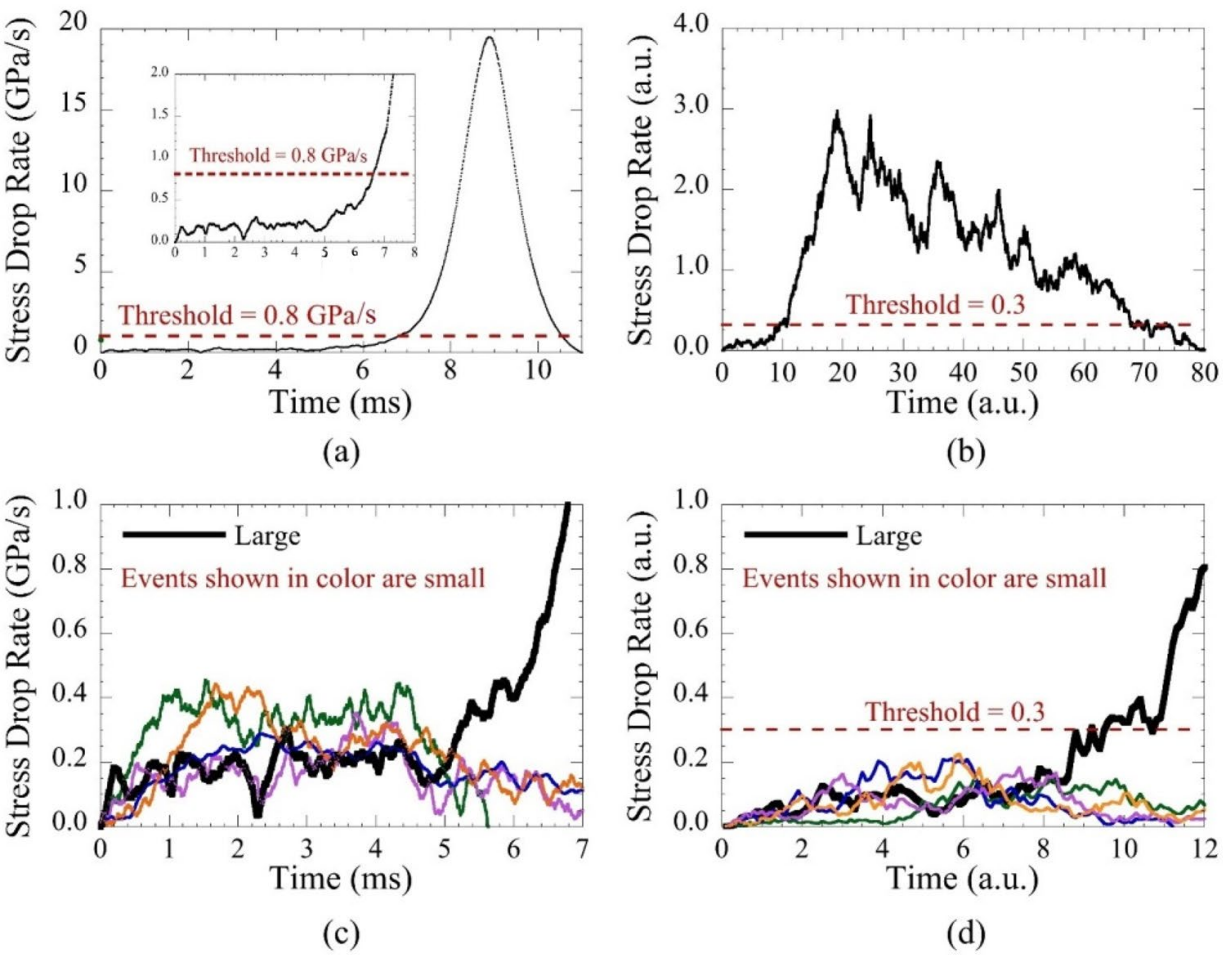
Experiment and simulation: The negative time derivative of stress as a function of time during large avalanches in Zr_45_Hf_12_Nb_5_Cu_15.4_Ni_12.6_Al_10_ BMG (**a**) and in mean-field simulations (**b**). The dashed line is the threshold below which the “foot” or nucleation event of the large avalanche can be seen. Inset shows a magnification of the same foot. The same feet are shown plotted with small events with size within ± 5% of the respective foot for BMG (**c**) and simulations (**d**). The feet are shown in black; the small avalanches are shown in color. The feet are indistinguishable from their small events until the nucleation event.

The data analysis uses established methods^1^. Instrument noise is minimized using Wiener filtering, and slip avalanches are identified as temporally connected regions of negative time derivatives of the stress (i.e. stress drops)^1^. Stress drops correspond to slip avalanches. These slip avalanches (or serrations) are the manifestation of shear band propagation. The negative time derivative of the stress (which is the stress-drop rate) for an avalanche event is shown in Fig. 2a. Its statistical properties provide detailed insight into the propagation dynamics. The exceptionally high time resolution and low noise of the experimental data enable identification of not only the large slips but also small slips, that can be up to 100 times smaller in size.

Remarkably, most large slip avalanches show a slow, jerky period before a fast, smooth period in the time profile, see Fig. 2a. We call the jerky region a “foot.” We interpret the foot as the nucleation phase of the large avalanche. These periods, or “feet,” are seen in 89% of the large avalanches that we observed. However we have never observed them in the last large event, which is the actual failure event during which the sample fractures. Most importantly, the feet appear to have the same propagation dynamics as the small avalanches. The same foot as shown in Fig. 2a appears in Fig. 2c, plotted with the stress-drop rates of small events. The stress-drop rate during the foot remains in the range of the small events throughout its duration without any distinguishing features until the dynamics of the large event changes at 5 ms. Both small events and the feet propagate slowly and in a jerky fashion. The data indicate that when a small avalanche starts, it is impossible to know whether or not it will eventually turn into a large avalanche. Only after it reaches a critical nucleation size can it then transition to the dynamics of a large avalanche that propagates much faster.

In other words, the feet that become large avalanches are statistically and dynamically indistinguishable from the small avalanches until the foot ends and the dynamics transitions to a smooth slip propagation with a steep increase in the stress-drop rate.

A foot is defined as the section of the avalanche before the stress-drop rate (henceforth also referred to as “velocity”) increases above a certain threshold. The value of the threshold is set to the maximum stress-drop rate attained during small events, 0.8 GPa/s. We only consider feet that last longer than 2 ms, which is the shortest observed slip-avalanche duration. A foot’s duration is simply the difference between the time at the beginning and the end of that foot, and a foot’s size is the difference between the stress at the beginning and the end. The increase in the stress-drop rate at the end of a foot is usually coincident with a burst of acoustic emission, see Fig. 6 of^2^, which we attribute to the onset of the fast shear for the fully formed large system-spanning shear band^2^ as the peaks in acoustic emission correspond to the timing of the nucleation.

## Possible causes

We now compare the data to different possible scenarios for the formation mechanisms of large events:

### Percolation

**Figure 3:**
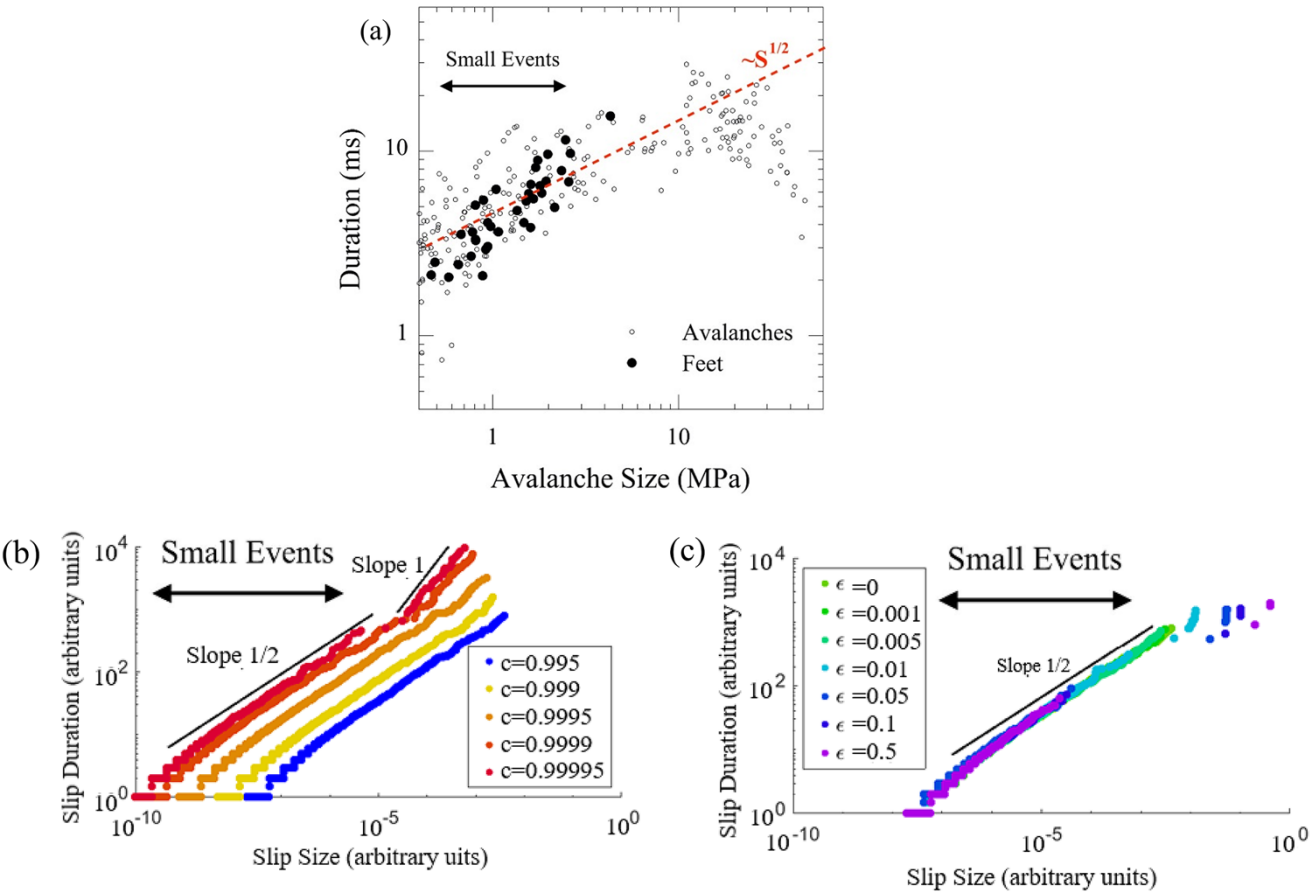
Experiment: (**a**) Duration as a function of size for small avalanches and for the feet of large avalanches in a Zr_45_Hf_12_Nb_5_Cu_15.4_Ni_12.6_Al_10_ BMG. The statistics of the two are indistinguishable in this plot. Simulation: (**b**) Duration as a function of size for simulations of small avalanches at a range of machine stiffnesses. The durations of large avalanches scale linearly with the size of the events. This is in contrast to the observed behavior in (**a**) in which the duration of large events does not increase and may in fact decrease with size. Simulation: (**c**) Duration as a function of size for simulations of small and large avalanches for various weakenings $\epsilon$ showing behavior similar to that in (**a**). Together (**b**) and (**c**) suggest that finite stiffness of the experimental apparatus is not the cause of the behavior of the large events, but rather that weakening is. Simulations are performed adiabatically with a system size of 10^5^ cells, ε = 0 and c = 0.996 except where specified.

In percolation, small avalanches link together to form a large avalanche. Accordingly, percolation requires that the avalanche duration *T* should grow linearly with size *S* as the small avalanches link up, *S* ~ *T*. However, we eliminate percolation as a possible scenario for the formation of large events because the duration *T* of the avalanches in fact follows the behavior *T* ~ *S*^1/2^ for small events and an even weaker dependence for large events, as shown in Fig. 3. Therefore, we rule out the possibility that large avalanches are simply merged small ones.

## Finite machine stiffness

An ideal mechanical test system would use an infinitely stiff machine to deform a sample of finite stiffness^28^. As the machine stiffness increases relative to the sample stiffness, the rate of decrease in stress for a given increment of plastic strain during the propagation of a shear band is greater for a stiffer system than for a more compliant system, thereby relieving the driving force for shear band propagation more effectively and enhancing the flow stability^29^.

The machine stiffness denoted as “low” means that it is “low” in relation to the sample stiffness. Our model simulations show that, in the case where the machine stiffness is too low, large avalanches form; however, the dynamics of these simulated large avalanches does not resemble the dynamics of the large avalanches seen in experiments. For insufficient machine stiffness, many large avalanches of similar sizes and durations merge together, leading the avalanche duration *T* to scale linearly with the avalanche size *S*, i.e. *T* ~ *S*, as seen in Fig. 3b. Also the characteristic shape of merged avalanches is fundamentally different from that of a single system-spanning avalanche. Since the model simulations for low stiffnesses show neither the observed dynamics nor observed scaling behavior for the large avalanches in our experiments, as in Fig. 3a, we can rule out a too-compliant machine as the cause for the formation of large slips.

### Finite size effects

One might wonder if the large avalanches are simply finite size effects that would perhaps disappear for sufficiently large samples. The answer is no. Finite size effects and stiffness effects are very closely related in this case. In the following we explain this in more detail.

The total combined stiffness of the sample and machine is K_total_. We treat the machine (with machine stiffness K_M_) and the sample (with sample stiffness K_S_) as two springs connected in series. The resulting total stiffness K_total_ is then given by K_total_ $\approx$ (1/K_M_ + 1/K_S_)^–1^ = (K_M_ K_S_)/(K_M_ + K_S_) $\le$ K_M._ Here the sample stiffness K_S_ = EA/L (with L $\ge \sqrt{A} )$ depends on the elastic modulus E and the ratio A/L of the sample, where A is the cross-sectional area and L is the length of the sample. In the experiments L = 3 $\sqrt{A}$.

At a critical value of the total stiffness K_total_^critical^ $\equiv$ K_L_ $\sim$ E $\surd \mathrm{A}$, the avalanche size distribution follows a pure power law over the entire size range up to the largest avalanches that intersect opposite boundaries of the sample^5,30,31^. We can then discuss two cases:

- The machine stiffness is lower than the critical stiffness K_L_, i.e. K_M_ < K_L_. In this case, the total stiffness K_total_ also stays below the critical value K_L_, because K_total_ $\le$ K_M_ < K_L_. In this case then, the large events are simply caused by the low machine stiffness effects discussed above, with a predicted scaling behavior of *T* ~ *S* for the largest events. This scenario is already ruled out by the fact that *T* ~ *S* does not hold for the largest events in the experiments.
- The machine stiffness is larger than the critical stiffness K_L_, *i.e*. K_M_ > K_L_. In this case the sample dimensions determine whether or not the total stiffness K_total_ is above or below K_L._ For K_total_ > K_L_ the size distribution of events is expected to drop off exponentially at an upper cutoff of the power law scaling regime that corresponds to (microscopic) slips that do not span the cross-sectional area of the sample^5,30,31^. Also in this case the dynamics of the largest avalanches is the same as that of the smaller avalanches in the power law regime. This case does not apply to the experiments shown here. The reason is that the experiments here have many more large (i.e. system-spanning) events than would be obtained if the distribution was a power law with a mere exponential cutoff at the upper edge of the scaling regime. Also the dynamics of the large avalanches in the experiments are markedly different from those of smaller avalanches in the power law scaling regime. Tuning the sample dimensions, however, changes K_S,_ and thus is a tool that could be used to tune the total stiffness K_total_ below the critical stiffness K_L_, i.e. K_total_ $\le$ K_L_. In that case one expects the same results as in the first scenario with the machine stiffness below K_L_, which also resulted in K_total_ $\le$ K_L_. But if that were the case, then the above scaling of *T* ~ *S*, would be expected even for the largest events. Since that is not observed in the experiments we can rule out finite sample size effects or equivalently too low total stiffness K_total_ $\le$ K_L_ as the origin of the large events in the experiments.

### Nucleation

Mean-field theory predicts that the avalanche dynamics changes when a small avalanche exceeds a microscopic critical slip size and spontaneously transitions to a large avalanche that extends from one side of the specimen to another. While the avalanche is still smaller than the critical size, the propagation dynamics is jerky, with many starts and stops (i.e. pulse-like) and distinctly different from the smooth simultaneous (i.e. crack-like) propagation dynamics during the large event^5^. This prediction for nucleation matches all of the phenomena seen in experiments where large avalanches have jerky “feet” that appear identical to the small-avalanche propagation dynamics during the incubation phase. In other words, an avalanche does not “know” whether it will become a large avalanche until it exceeds the critical size for nucleation.

Evidence of the feet can also be observed in displacement data for large system-spanning shear bands obtained using high-speed imaging^27^. Digital image correlation shows a period of slowly increasing displacement (i.e. the foot) followed by an increase in the displacement rate that is coincident with a rapid increase in the stress-drop rate. The increase in the stress-drop rate is usually coincident with a burst of acoustic emission, which we attribute to the onset of the fast shear for the fully formed large system-spanning shear band^2^.

The mean-field model predicts that the critical size for nucleation and thus the feet should get smaller for larger weakening^5^. We expect the largest weakening to occur at the fracture event when the shear band has widened the most. We indeed find that the duration of the feet of all the smaller events is much larger than experimental observations of the total duration of fracture events^27^. This suggests the weakening at failure is so large that the foot size is negligibly small. In other words, the absence of a foot in the experimental data is consistent with the notion that fracture may be modeled as 100% weakening, corresponding to a vanishing foot size^5^.

## Simulations

We have performed mean-field simulations that include threshold weakening^5,30^ (defined below), and find feet in the simulated large avalanches. The mean field theory (MFT) model assumes elastically coupled weak spots slip by a random amount when the local stress exceeds a local stress threshold. Because of the elastic coupling, a slipping weak spot can trigger others to slip also giving rise to a slip avalanche, which is observed as a shear band in a BMG. The weakening is implemented as a temporary lowering of the threshold of each weak spot after it slips. The weakening lasts until the end of the slip avalanche. (For details on the model, see the Supplementary Material.)

The simulated feet statistics are indeed consistent with the experimental observations and predictions from MFT. A foot from simulations at finite weakening is shown in Fig. 2b, d. Furthermore, comparisons between these and the experimental results in Fig. 2a, c show the strong resemblance between the feet in BMGs and those in simulations. The experimental sizes and durations of all feet and all small avalanches in our BMG are plotted in Fig. 3. All of the feet are well within the range of sizes and durations for small avalanches as predicted by the model. Slip models that do not incorporate a weakening mechanism would not predict the occurrence of feet.

To better understand the weakening, we can use the simulations to examine the local stress distribution at the start and end of the avalanche feet. We can consider three regimes of the local stress τ, normalized by the failure stress τ_c_, so that failure of a cell (i.e. local slip) occurs when τ/τ_c_ is 1 or higher. These three regimes are (1) below the initial unweakened failure stress (0.96 < τ/τ_c_ < 0.98), (2) near the failure stress (0.98 < τ/τ_c_ < 1), and (3) failing (τ/τ_c_ ≥ 1). The percentages of cells at those stresses are shown in Table 1 to illustrate the relative distribution of stress throughout the system.

**Table 1:**
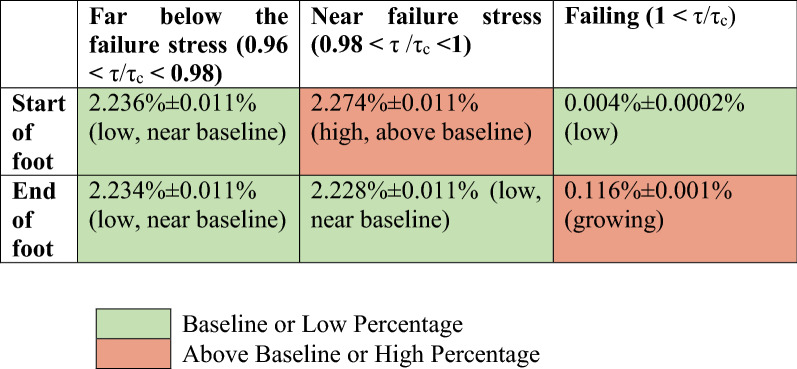
Percentage of cells at various normalized stress levels τ/τ_c_ before and after the avalanche foot (which is the time during which the nucleus of the large avalanche reaches the critical size to initiate a system-spanning event). All measured events occur after all transients from the initial conditions have subsided. Confidence intervals for the error bars are 2σ. System size = 100,000 cells, weakening = 0.007, stress conservation parameter (see SI and^5^) c = 0.997, number of time steps = 100,000. The baseline is defined as 2.23%. The table illustrates how a stress concentration (i.e. an unusually high number of cells synchronized near the local failure stress) can result in the nucleation of a large event. Note that for any cell, τ/τ_c_ can jump in one discrete time step from τ/τ_c_ < 1 to τ /τ_c_ > 1 and vice versa.

The percentage of cells that have a stress far below the failure stress is nominally the same at the beginning and end of a foot, 2.236% vs. 2.234%. The key differences in the stress distribution of cells between the start and end of the foot are seen in the percentage of cells near failure and the percentage of cells that are actually failing. At the beginning of the foot, the percentage of cells near failure is relatively high (indicating stress synchronization and that the system-spanning avalanche is about to commence), while the percentage of actually failing cells is close to zero, similar to small avalanches. This fact reflects that the system-spanning part of the avalanche has not yet started. When the foot has ended (i.e. a small avalanche has grown sufficiently large), the stress distribution has shifted: even though the percentage of cells near failure is relatively low, the percentage of failing cells is high. This is because the stress concentrated cells have failed synchronously, as a group, causing many cells to rapidly fail and nucleating the otherwise small event into a large one. The mechanism is akin to crack nucleation where a stress concentration synchronizes cells at the crack boundary to all be close to failure. When the cells fail and continue to fail due to the loss of cohesion in a crack, the increase in stress at the crack boundary is so large that more cells fail and continue to fail. The difference between shear bands and cracks is that in shear bands, cohesion is maintained (though weakened), while a crack opens and typically has no cohesion. Again see Table 1 for the numerical details of the stress distribution. “Stress concentrations,” where the number of cells is high or above the baseline, are shown in red.

**Figure 4:**
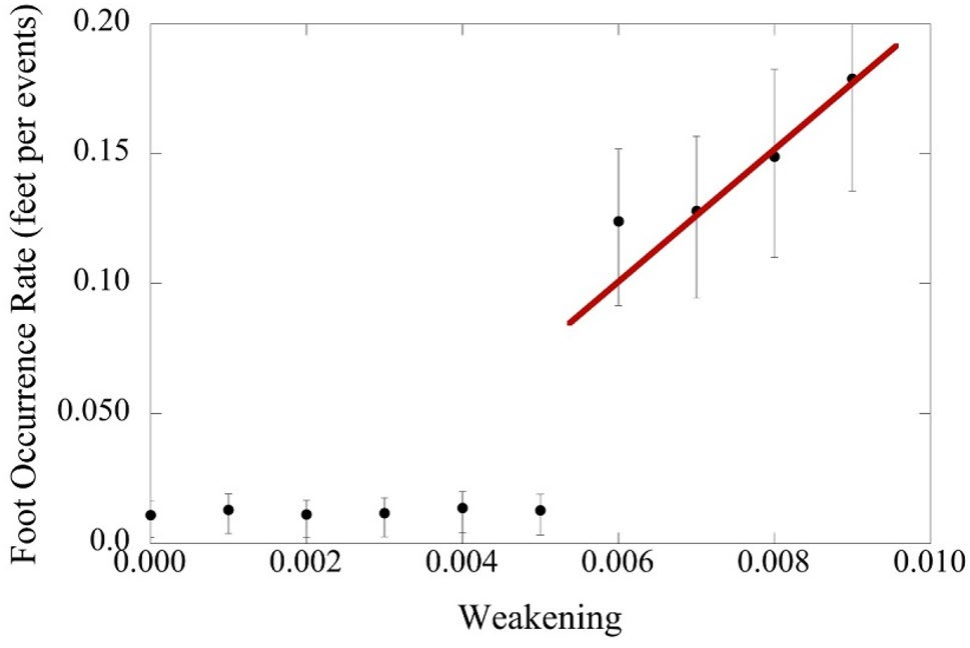
Simulation: The foot occurrence rate as a function of weakening in the mean-field simulations for large avalanches that have a size > 10^−6^ in simulation units. The rate is defined as the fraction of avalanches that are larger than 10^–6^ that have a foot. For weakening above ~ 0.006, large, system-spanning events become prevalent, and the foot rate increases approximately linearly with weakening. System size = 10^5^ cells; *c* = 0.9986; 95% error bars are shown.

Weakening has, until now, been discussed only qualitatively. As the nucleation of large events from feet is a direct consequence of weakening, the nucleation sizes allow for quantitative insight into the effects of weakening in a system. In Fig. 4, we show the fraction of the total number of events (small and large together) that exhibit a foot. We call this fraction *the foot occurrence rate*. Physically one could think of this rate as the ratio of large events (formed by the nucleation mechanism) relative to all events in a simulation or experiment. Figure 4 shows the foot occurrence rate as a function of weakening. As mentioned above, the nucleation size for large events decreases with increasing weakening. Therefore we see a step function increase in the foot occurrence rate at the weakening that corresponds to nucleation sizes that are equal to the largest events within the scaling regime, allowing small events to nucleate into large ones. This occurs at roughly ε = 0.006 in our simulations. Physically this situation corresponds to materials that have sufficient weakening to form system-spanning shear bands for a given sample size. (Note that this value of weakening is system-size dependent, and conversely, the smallest sample size above which system spanning shear bands occur is weakening dependent—the larger the weakening the smaller the smallest system above which these system spanning shear bands are formed). Once this weakening is exceeded, the foot occurrence rate increases linearly with weakening since the fraction of large events grows with the weakening and only the large events have feet. Unlike weakening, the foot occurrence rate (or equivalently the number of nucleated large events divided by the number of all events) is easily measured in experimental systems with sufficiently high temporal resolution, and thus may serve as a quantitative probe for the weakening in that system.

The foot occurrence rate may change based on factors that change the deformation properties of the system such as system composition, size, strain rate^32^, and temperature^33^. If so, the foot occurrence rate may not be comparable between very different systems, but between similar systems, it may serve as a direct comparison of the weakenings of those systems. This allows us to compare different materials directly and to quickly order them according to brittleness. Materials with a greater foot occurrence rate (or equivalently with a greater fraction of large, i.e. system-spanning events) have greater weakening, and are thus expected to be more brittle.

The foot rate as presented here provides a *dynamical* method for identifying the weakening through the linear relationship between the weakening and the foot occurrence rate. Because the foot-rate method identifies large events not just from their size but also from their dynamics, it is able to distinguish between situations where the large events are truly reflecting the weakening in the material and situations where they are resulting merely from machine or sample stiffnesses that are too low. If there is a large gap between the upper cutoff of the power law scaling regime of the slip size distribution and the sizes of the largest events, then we can conclude that the results of the foot rate (or of the other methods) are not skewed by finite size effects, because in that case the largest avalanche size in the power law scaling regime is far below the system size, so that small avalanches and large system-spanning avalanches are well separated in size.

It has been previously observed that the introduction of crystalline dendrites into the BMG may mitigate the formation of large avalanches^34^ and increase ductility^35^. Interestingly, in^34^ the observed foot occurrence rate differs greatly between the monolithic metallic glass, 89%, and the composite, 19%. This difference suggests that the introduction of the crystallites inhibits the nucleation of large events, likely by decreasing the weakening in the system. It furthermore shows the utility of the foot occurrence rate as a tool for the characterization and comparison of deformation mechanisms in different materials. We similarly expect that the foot occurrence rate would differ between an as-cast BMG and a structurally rejuvenated one^36,37^ due to the difference in the degree of disorder.

Until now, the weakening could only be measured indirectly by comparing avalanche size distributions of different materials, or by comparing the fraction of all avalanches that are large events. These methods give mostly qualitative estimates of the weakening that can be skewed by finite size and resolution effects. The foot-rate method provides deeper insight into the dynamics of the deformation mechanism than statistical methods alone can do. Using both the statistical methods and the foot-rate method in concert should give a consistent and much more complete understanding of the underlying deformation mechanism because the feet provide access to the weakening as a fundamental materials property. With this characterization, we can explicitly compare different materials. The prediction of the model is that more brittle materials have a higher foot rate, and thus higher weakening, than less brittle materials.

We note the qualitative similarity between this process and other processes such as an avalanche oscillator^38^, in which relaxation of the edge dislocations leads the system to “oscillate” close to and away from criticality, causing large events to form quasi-periodically. Both mechanisms lead to approximately periodic large events. Like an avalanche oscillator, stress concentration causes the past behavior of the system in question to affect the size and distribution of future events. However, unlike an avalanche oscillator, the process of stress concentration may be self-reinforcing as large events may themselves cause stress concentrations through multiple failures of weakened cells during the same event, thus stress synchronizing those cells as well. Also the avalanche oscillator relies on the presence of some kind of hardening mechanism, while the stress concentration mechanism does not.

Ductility will be increased when the critical nucleation size for large shear bands cannot be attained because in those cases, large system-spanning shear bands simply will not form. Evidence for this notion can be seen in the observations of Conner et al. who found that Vitreloy 106 metallic glass wires with diameters below a certain size show enhanced ductility^39,40^. We approximate this spatial dimension by comparing the stress drop sizes of both the large events and the feet. We note that the nucleation size in units of stress is approximately one order of magnitude less than the stress drop size of the largest events. The largest events span the entire shear plane. Assuming a linear relationship between stress drop size and slip area, we would then expect the cross-sectional area of the nucleation size to be approximately one order of magnitude smaller than the area of the shear plane. Although this relationship is not truly linear for the large events as cells may fail multiple times, such effects do not grow the stress drop more than an order of magnitude. We therefore estimate the critical nucleation size to be on the order of 10^–1^ mm^2^ using the experimental data shown here, which is approximately equal to the wire cross-section below which ductility is known to increase substantially^39,40^. In this case, the restriction on the critical nucleation size imposed by the system size in combination with the inhomogeneous stress state due to bending leads to increased plasticity. Initially, large shear bands do not form, although small ones still will, because the critical nucleation size is larger than the wire diameter. As the bending strain increases, the weakening also increases as tension increases dilation, causing the critical nucleation size to decrease, ultimately leading to the formation of shear bands associated with large avalanches. We note that predictions for the ductility of specimens with nanoscale and microscale dimensions require separate consideration as demonstrated by^37,41^. For deformation experiments involving metallic glass specimens with nano- and microscale dimensions, see^42–48^.

We have demonstrated that the sample size below which a sample becomes ductile can be predicted from the slip statistics seen at larger sample sizes. Our findings also have implications for strategies to increase the ductility of BMGs. Ductility can be improved by preventing the nucleation of the largest slips. This can be achieved either sufficiently reducing the sample size or by sufficiently increasing the total stiffness K_total_ or by engineering the microstructure (through appropriate spacing of heterogeneities) such that the largest slips remain smaller than the nucleation size. Naturally occurring heterogeneities (*e.g.* porosity) could also effectively reduce the weakening, so as to sufficiently increase the nucleation size to achieve the same effect.

In summary, we have identified the mechanism of system-spanning shear bands in BMGs in both our experiments and simulations. The end of the foot in the stress-drop rate – time profile of a large avalanche corresponds to a small avalanche that nucleates into a large system-spanning shear band, thereby marking a transition in the propagation dynamics of the avalanche. We have devised a method to quantitatively identify these feet in experiments and simulations. The predictions of a simple mean-field model explain the observations, including the observation that the final failure event has no foot (presumably dilation has increased the weakening to the point that the nucleation size becomes infinitesimal). The agreement between the experiments and the mean-field model further extends the notion of universality in plasticity. For applications beyond BMGs, the analysis also provides a method to identify the cause for large events observed in experiments or simulations and to distinguish between the different causes, with event nucleation as one possibility and percolation, finite size effects, or too low machine stiffness as others. Finally the study provides a way to use the slip statistics in macroscopic samples to predict the sample size below which a material becomes more ductile. The model also predicts ways to increase ductility in new materials and to rapidly test for relative ductility in libraries of new BMG compositions.

## Supplementary Information

Supplementary Information.

## Data Availability

All data used in this study is available from the corresponding author upon reasonable request.
