# Supplementary material for: Experimental evidence that shear bands in metallic glasses nucleate like cracks
Source: Sci Rep. 2022 Nov 2;12:18499. doi: 10.1038/s41598-022-22548-8 (PMC9630419; doi:10.1038/s41598-022-22548-8)
Supplement: Supplementary file 1 — Supplementary Information. [file 41598_2022_22548_MOESM1_ESM.docx]

**Mean-field model:** The premise of the mean-field model is that materials deform via slipping weak spots. In the mean-field model, a shear band is divided into *N* cells, each of which can slip when the local shear stress exceeds a local failure threshold [36], *i.e.* weak spots slip first. The released stress is redistributed to the other cells via long-range interactions. In the parlance of the mean-field model, each slipping event is known as an “avalanche.” A weak spot slips, causing other weak spots to slip, thereby causing a slip avalanche. This slip avalanche manifests as a serration in mechanical test data that may or may not be observable depending on the resolution of the measurement device.

The mathematical description of the mean-field model is as follows. The shear band is divided into N cells. The local shear stress *τ_l_* at cell *l =* 1 , … , *N* is given by

τ*_l_ = J*/*N* Σ*_m_* (*u_m_ – u_l_*) *+ F***,**

where *F* is the applied shear stress, *u_l_* is the local displacement discontinuity across the shear band**,** and *J* is the elastic mean-field coupling. The sum runs over all cells *m =* 1, … , *N*. The local failure stress is *τ_f,l_ ≡ τ_s,l_* and if there is weakening then the local failure stress reduces to τ*_f,l_≡*τ*_d,l_*< τ*_s,l_*. The weakening can be quantified by a weakening parameter ε*≡*(τ*_s,l_ –*τ*_d,l_* )/(τ*_s,l_*– τ*_a,l_* ) with a sticking stress τ*_a,l_* < τ*_d,l ._* When cell *l* fails, it slips by a (random) amount Δ*u_l_*. The local stress is reduced to the local sticking stress τ*_a,l_* < τ*_d,l,_ chosen from a random distribution of width r*, and the local stress drop is given by τ*_f,l_ –*τ*_a,l_~*2*G*Δ*u_l_* where *G ~ J* is the elastic shear modulus. After the slip, the cell resticks. The released stress is redistributed to the other cells in the system, which can then trigger other sites to slip in a slip avalanche. The avalanche ends when at all sites the local stresses are below their local failure stresses τ*_l_<* τ*_f,l_.* In the adiabatic (slow driving) limit, the applied stress *F* is increased only after a slip avalanche has been completed. It is then increased until the next cell fails. If the system is driven by a small, imposed strain rate Ω*,* rather than a slowly increasing applied stress, then the stress *F* in the above equation of motion is replaced by *K_L_*(Ω*t – u_i_*) where *K_L_* is an effective loading spring constant [5,7], which is proportional to $G/\surd N$.

The weakening ε is critical to the limited ductility of BMGs. For BMGs the most likely weakening mechanism is local dilation caused by a slip. As the system is deformed, the stress on each cell increases up to a local failure stress. When this stress is reached, the cell slips by a random amount, releasing its stress to the rest of the system. When weakening is present, the local failure stress is reduced to a lower (weaker) value than before a slip [27]. After each avalanche, we assume that the thresholds reheal to their original strengths.

For nonzero threshold weakening (ε > 0) as is the case for BMGs, the model predicts two types of slip avalanches: small avalanches and large avalanches, which have different statistics and dynamics, just like the small and large shear band events. The small avalanches are microscopic in size and have a power law size distribution. They propagate progressively, in a jerky fashion with sudden accelerations and decelerations. In contrast, the large avalanches are macroscopic in size, cutting through the entire specimen, and typically have a narrowly peaked size distribution for large weakening. They propagate simultaneously, with first a smooth increase and then a decrease in the avalanche propagation speed. Our experiments on BMGs confirm all of the model predictions that we have been able to test thus far [1].

**Foot Occurrence Rate Threshold Size:**

Figure S1 is a plot of the foot occurrence rate as a function of weakening according to simulations for avalanches that have a foot larger than a certain threshold size (*i.e.* 10^–3^, 10^–4^, etc. in simulation units). For experimental data, such a threshold size could be a way to exclude potentially false small events that may result from experimental noise. We see that the linear relationship between the foot occurrence rate and the weakening is retained for threshold sizes orders of magnitude larger than the smallest avalanche events. This result demonstrates that this method to use the foot occurrence rate to quickly order materials according to weakening (or brittleness) can be applied to experimental systems that do not have sufficient resolution to see the smallest events. We note that for foot rates with threshold sizes larger than 10^–3^ almost all included avalanches are system-spanning avalanches so that the foot occurrence rate is near its saturation value of 1 and thus the linear dependence on weakening is lost.

**
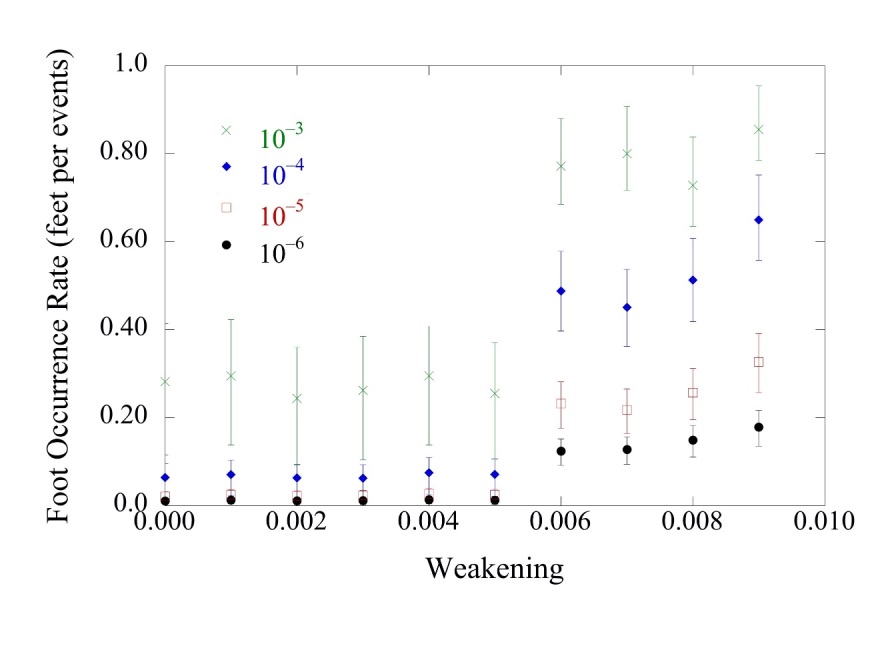
**

**Figure S1. Simulation:** The foot occurrence rate as a function of weakening in the mean-field simulations for large avalanches. System size = 10^5^ cells; c = 0.9986; 95% error bars are shown.
